# Foreign aid and the rule of law: Institutional diffusion versus legal reach

**DOI:** 10.1111/1468-4446.12752

**Published:** 2020-04-14

**Authors:** Andrew Dawson, Liam Swiss

**Affiliations:** ^1^ Department of Sociology York University, Glendon Campus Toronto Canada; ^2^ Department of Sociology Memorial University St. John’s Canada

**Keywords:** development, foreign aid, institutions, norms, rule of law, security sector reform

## Abstract

This paper examines the role of bilateral foreign aid in supporting the diffusion and enactment of common models and institutions of the rule of law among aid‐recipient low‐ and middle‐income countries. We ask whether aid targeted at security‐sector reform and the rule of law influences the adoption of constitutional and legal reforms over time (institutional diffusion), and whether aid also supports more effective implementation of the rule of law, writ large (legal reach). We use event history and fixed‐effects panel regression models to examine a sample of 154 countries between 1995 and 2013 to answer these questions. Our findings suggest that aid does increase the likelihood of adopting several rule of law reforms, but its effect on increasing the depth or quality of rule of law over time within countries is much less substantial. These findings suggest that though aid may play a role in supporting the diffusion of models contributing to state isomorphism among countries, it is less effective at increasing the pervasiveness and quality of such model’s implementation. This discrepancy between the effectiveness of bilateral aid in promoting law on the books versus law in action in aid recipient countries calls into question the current approach to rule of law reforms.

## 1 INTRODUCTION

Why do states share common approaches to the rule of law and the security sector, despite diverse individual histories and contexts? World Society Theory explanations for this isomorphism argue that this reflects the influence of international organizations, networks of legal and security sector experts, and a common world cultural model for how a legitimate state should structure legal institutions and promote the rule of law throughout society.

Despite this, the rule of law has received less attention in the World Society research literature than many related but distinct issues like human rights. Apart from a few examinations of the diffusion of specific types of legislation for sex crimes (Frank, Camp, & Boutcher, 2010; Frank, Hardinge, & Wosick‐Correa, 2009), abortion (Boyle, Kim, & Longhofer, 2015), and terrorism (Shor, 2011, 2017), legal reforms have seldom been examined through a World Society lens. Similarly, while there are a few cross‐national studies examining the influence of foreign aid on the rule of law (Knack, 2001; Rajan & Subramanian, 2007; Young & Sheehan, 2014), none assess the effect of aid specifically earmarked for the rule of law.

In this paper, therefore, we take up Swiss’s (2016c) call to look more closely at the role of foreign aid in the diffusion of world society norms/models on a sector‐by‐sector basis. In doing so, we ask whether foreign aid targeted at security sector reform and legal/judicial reform influences both: (a) the diffusion of common rule of law norms and institutions among countries; and (b) changes in the level of the rule of law within societies over time.

We examine these questions through a two‐stage research methodology. First, we use event history analysis to examine the diffusion of constitutional and legal reforms in low‐ and middle‐income aid‐recipient countries. Second, we use instrumental variable two‐way fixed‐effects panel regression to explore the effects of aid on the changing degree of the rule of law within aid receiving countries over time. We examine a sample of 154 aid‐recipient countries between 1995 and 2013.

Our findings suggest that aid, generally, is associated with the diffusion of and legal and constitutional reforms associated with the rule of law. More specifically, different categories of aid (from more general to more specific) have different effects, depending on the type of legal/constitutional reform analyzed. Moreover, our results show that while total aid has a negligible effect on the degree of the rule of law measured in a variety of ways, more targeted measures of aid—particularly security sector reform aid—show a beneficial, albeit minor, effect on select measures of the rule of law. We conclude, therefore, that aid’s role in the spread and institutionalization of world society models of the rule of law appears more consequential to the diffusion process of state institutional structures than to its impact on rule of law outcomes. The discrepancy between the two points to limitations of the current approach to rule of law reforms.

### 1.1 Conceptualizing the rule of law

While acknowledging that the rule of law is a contested concept (see Costa, Zolo, & Santoro, 2007; Tamanaha, 2004), consistent with a World Society approach, we analyze it along two dimensions—the formal legal apparatus and the reach of the law throughout society. The distinction between the two dimensions of the rule of law is roughly analogous to the classic distinction between “law on the books” and “law in action” (see Halliday & Carruthers, 2007).

First, the rule of law necessitates a legal system that enforces established rules of conduct (i.e., laws). This dimension of the rule of law therefore pertains to the legal apparatus at the state level, with a focus on constitutional frameworks, legal institutions, and/or individual laws. As described below, we measure change in this dimension of the rule of law by the adoption of constitutional and legal reforms.

The second dimension of the rule of law refers to “the authority and influence of law in society, especially when viewed as a constraint on … behaviour” (as cited in Frenkel, 2017). That is, it is the capacity of the law to effectively permeate society and influence conduct or, in other words, the “infrastructural power” of the state’s legal system (Mann, 1984). The focus of this dimension is therefore on the society‐wide diffusion of the law. Notably, this focus excludes an analysis of the extent to which the government’s power respects meaningful legal constraints. While limits on government power are a fundamental element of the rule of law, our interest here lies in the influence of the law beyond the state. This dimension of the rule of law therefore corresponds to the concept of “rule by law” (also known as “law and order”)—that is, the ability of the law to serve the power of the state but not to limit it (Carothers, 1998). While more difficult to operationalize, this dimension of the rule of law can be measured by the degree of legal compliance among the population (see Weber, 1968) and/or widely held attitudes toward, or perceptions of, the legal order, including the level of popular confidence in the legal system and whether the law is considered to be impartially and uniformly applied.

## 2 LITERATURE REVIEW

### 2.1 World society and the rule of law

The World Society perspective of sociological institutionalism aims to explain the striking isomorphism we see across states and societies (Boli & Thomas, 1999; Lechner & Boli, 2005; Meyer, 2007, 2009; Meyer, Boli, Thomas, & Ramirez, 1997). Centered on the work of John Meyer and his many students and collaborators, this perspective explains isomorphism as arising from the enactment of common models, norms, and policies by states and other organizations in an effort to achieve legitimacy (Meyer, Boli, et al., 1997). These models, policies, and norms make up a world culture that is promulgated and refined through the efforts of the “rationalized actors” of World Society: international organizations, networks, professionals, scientists, and so on.

Research in this perspective has most often focused on explaining, therefore, the diffusion of these models and the connection of states to the actors and networks of World Society. Research has shown the spread of world cultural models linked to issues including, but not limited to: human rights (Clark, 2010; Cole, 2005, 2012a, 2012b; Cole & Ramirez, 2013), education (Schofer & Meyer, 2005), environmental institutions (Frank, Hironaka, & Schofer, 2000; Frank, Longhofer, & Schofer, 2007; Longhofer & Schofer, 2010; Meyer, Frank, Hironaka, Schofer, & Tuma, 1997; Schofer & Hironaka, 2005), women’s empowerment and gender equality (Berkovitch, 1999; Cole, 2013; Nugent & Shandra, 2009; Swiss, 2009; Wotipka & Ramirez, 2008; Yoo, 2011), corporate policy (Lim & Tsutsui, 2012), and legal reforms (Beck, Drori, & Meyer, 2012; Boyle et al., 2015; Frank et al., 2009, 2010). The general conclusion of much of this research has been that the more ties or connections a state has to the actors and networks that compose World Society, the more likely they are to adopt and enact various world cultural models and practices.

Research into the rule of law and World Society has been rather limited and primarily focused on the diffusion of certain types of liberalizing criminal legislation or constitutional reforms (see above), corporate bankruptcy laws (Halliday & Carruthers, 2007), the expansion of government structure (Kim, Jang, & Hwang, 2002) and relatively few studies of the diffusion of common security/policing institutions (Deflem, 2002; Eyre & Suchman, 1996; Hironaka, 2009; Jepperson, Wendt, & Katzenstein, 1996; Shor, 2017; Suchman & Eyre, 1992; Swiss, 2011). Like other World Society research, these studies generally support the idea that there are common models linked to security and the rule of law that have diffused across many country contexts and are expected institutional forms in legitimate states. Our research here examines this diffusion and the effects of aid on it.

### 2.2 Aid and the world society

Foreign aid’s role in funding the diffusion and institutionalization of world society norms has only recently become a focus of research (Fejerskov, 2015; Peterson, 2014; Swiss, 2016b, 2016c, 2017; Velasco, 2020). This research has shown that the more ties to bilateral aid donors that states have, the more likely a country is to adhere to world society human rights norms and join international organizations (Swiss, 2016b). For instance, Velasco (2020) shows that countries receiving aid from more donor countries, and those receiving more aid overall are predicted to offer more protections to LGBT rights. Likewise, bilateral aid donors are more likely to provide aid to countries which are already more embedded in international networks (Swiss, 2017) or with whom they share more common memberships in international organizations (Swiss & Longhofer, 2016). Other research has examined the dynamics of how aid donors are subject to and enact World Society norms and models (Brown & Swiss, 2013; Cold‐Ravnkilde, Engberg‐Pedersen, & Fejerskov, 2018; Engberg‐Pedersen, 2016, 2018; Swiss, 2011, 2012, 2018).

Swiss (2016b) argues that aid’s role in World Society is one of both diffusing models/norms and increasing the embeddedness of states in global networks which promote the same. By brokering connections between recipient‐country governments and organizations, aid funds help pay for the transfer of expertise and ideas that takes place in development assistance initiatives and may also work toward narrowing or closing decoupling gaps. Still, little is known about the sectoral efficacy of this role for aid. Much of the existing research has examined aid’s role at the macro level and there remains a gap in knowledge here about how aid focused on specific sectors influences World Society diffusion processes. As a result, our study looks at this sectoral focus using the rule of law as a case study.

### 2.3 Aid and the rule of law

**Figure 1:**
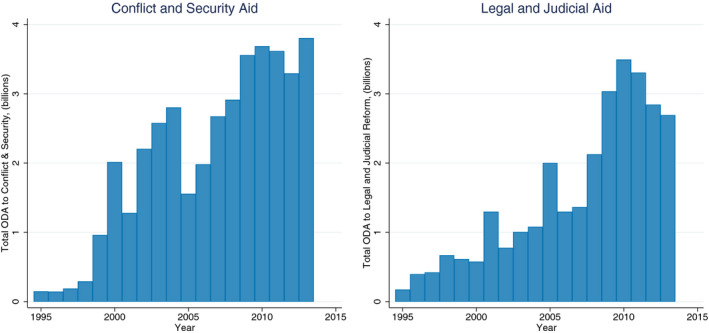
Total ODA to conflict and security and to legal and judicial reform by year, 1995–2013 [Colour figure can be viewed at wileyonlinelibrary.com]

The rule of law is often subsumed under the banner of good governance and is considered to be one of its central components. Good governance in recipient countries has been shown to attract development aid from some donors (In’airat, 2014; Neumayer, 2003), but also to have limited influence on the aid allocation of others despite political conditionalities (Furuoka, 2005; Hout, 2002). While the rule of law is acknowledged as an important correlate of various facets of development (Acemoglu, Johnson, & Robinson, 2001; Dam, 2006; Dawson, 2010; Diamond, 2008; Fukuyama, 2011; Kaufmann, Kraay, & Zoido‐Lobatón, 1999; North, 1990; Rigobon & Rodrik, 2005; Tebaldi & Mohan, 2010), efforts to strengthen the rule of law in certain countries are also undertaken with donors’ national security interests in mind. This has led to the securitization of aid in recent years, with rule of law development assistance becoming a key foreign aid priority (Brown & Grävingholt, 2016; Swiss, 2011, 2016a). Consequently, aid spending related to the rule of law has expanded significantly. As Figure 1 shows, since 2000, donors have dedicated over a billion dollars of official development assistance per year to conflict and security aid, and nearly as much to legal and judicial reform aid. Indeed, since 2004, the United States alone spends about US$1 billion each year on rule of law reform abroad (Kleinfeld, 2012).

The cross‐national literature suggests that aid, generally, is ineffective at promoting the rule of law. Indeed, Knack (2001), Rajan and Subramanian (2007), and Young and Sheehan (2014) all find that aid flows have a significant negative association with various measures of the rule of law—that is, aid is counterproductive and its effect is deleterious. However, these studies limit their evaluations to total official development assistance, and do not take into consideration the amount allocated to specific categories of aid related to the rule of law, namely those in Figure 1—conflict and security (including security sector reform) and legal and judicial reform. This distinction is of the utmost importance in assessing the effectiveness of official development assistance in promoting the rule of law (in addition to its role in diffusing rule of law institutions) as, on average, most official development assistance is allocated to other sectors (e.g., infrastructure, health, education) that are not expected to have a significant effect on the rule of law. Therefore, no large‐*N* cross‐national research has empirically examined the effectiveness of aid specifically dedicated to the rule of law.

Case study research, moreover, has directly investigated the effect of official development assistance targeted at strengthening the rule of law. In line with World Society explanations of state isomorphism, Cravo (2016, pp. 108, 119) finds that rule of law reforms in Georgia and security sector reform in Guinea‐Bissau were “informed and undergirded by a liberal ethos and a practice of expert knowledge,” prioritizing “the transplantation of a western liberal legal framework.” However, despite goals of broadly promoting the rule of law, she finds that in practice initial objectives were narrowed to a focus on legislative reform, ignoring its implementation—that is, the focus became one solely of “law on the books.” Cravo (2016, p. 119) argues that this narrow focus has proved inadequate in promoting security, resulting in a “stark gap between these formal amendments and the lived reality of insecurity faced by citizens on the ground.” Indeed, the EU had labeled their security sector reform intervention in Guinea–Bissau as a success after westernizing legislative reforms of the military, police, and justice sectors were passed, even though the security situation had worsened (and despite the coup attempt near the end of the EU’s security sector reform mission in 2010, resulting in the Armed Forces Chief of Staff being illegally and unconstitutionally detained). While this research provides important insight, the question of whether these two cases are anomalous or apply more generally remains open. As such, this study conducts a large‐scale cross‐national test of the broader applicability of these findings.

To undertake a sectoral focus of the influence of aid on World Society diffusion processes, we assess the effects of official development assistance on two dimensions of the rule of law—the adoption of constitutional and legal reforms and changes in the level of the rule of law. We examine the consequences of aid targeted at reforming and managing legal institutions (provided under the rubric of Security Sector Reform and Legal/Judicial Reforms) as the main area of aid spending with a specific focus on the rule of law and therefore the most likely contributor to the diffusion of rule of law norms and institutions. According to the Development Assistance Committee Handbook of the Organization for Economic Co‐operation and Development (OECD), Security Sector Reform refers to the reform of “a broad range of security and justice institutions” and the governance and oversight of these institutions (OECD, 2007, p. 5), while legal and judicial aid supports reforms to formal and informal aspects of the justice sector. While security sector reform and legal/judicial aid may not capture all rule of law reforms, they are the primary Development Assistance Committee foreign aid categories associated with these reforms.

Given this definition, it is evident that aid dedicated to security sector reform and legal/judicial reforms, along with most other forms of rule of law development assistance, primarily targets the first dimension of the rule of law in attempting to reform the legal system and to create or change laws and, in some cases, constitutions. Aid dedicated to rule of law reforms often does not explicitly or directly target the second dimension of the rule of law (partly because it is more difficult). However, at least implicitly, state‐level legal reforms are often not an end in themselves, but are pursued to promote order by affecting the authority and influence of the law within society. Indeed, an underlying assumption of these reform efforts is that the second dimension of the rule of law will be enhanced once the proper legal institutional framework is in place. As such, following Cravo (2016), it is important to not only assess whether aid that targets rule of law reform is successful in changing legal institutional frameworks at the state level, but also to examine the extent to which aid is related to changes in the society‐wide reach and influence of the law.

### 2.4 Hypotheses

Given this background on World Society, aid, and the diffusion of rule of law norms and institutions, we aim to test the influence of foreign aid in support of the rule of law. Our analysis below therefore tests the following hypotheses:

Hypothesis 1 Security sector reform aid and legal/judicial reform aid increases the likelihood of the adoption of constitutional and legal reforms in recipient countries.

Hypothesis 2 Security sector reform aid and legal/judicial reform aid strengthen the rule of law at the societal level within recipient countries.

Hypothesis 3 Security sector reform aid and legal/judicial reform aid have a stronger effect on diffusion processes and rule of law measures than broader categories of aid more generally.

## 3 METHODOLOGY

### 3.1 Approach

Our analysis proceeds in two stages. First, in analyzing the first dimension of the rule of law, we use event history analysis to model the effects of foreign aid on the adoption of three types of legal institutional reforms. Then, we model the effect of aid on four rule of law measures that capture the second dimension of the rule of law to assess the influence of development assistance on broad‐based changes in the levels of the rule of law within countries over time.

In Stage 1, event history models predict aid’s effect on the rate of occurrence of the institutional adoption or reforms we examine as representing an increased institutionalization of world society norms of rule of law. We estimate hazard ratios for each of our independent variables and controls using an exponential event history model (Box‐Steffensmeier & Jones, 1997). This model assumes that the hazard rate for each of the event types is constant throughout the period. Countries enter the analysis risk‐set in the first year of our sample period (1995) unless they became independent following that point, in which case they enter the risk‐set in their year of independence. Because each of our events are repeatable, countries only exit the risk‐set at the end of the period in 2013, and re‐enter the risk‐set following the occurrence of an event (Box‐Steffensmeier & Zorn, 2002). The details of the event data are outlined below. This approach in Stage 1 of the research lets us assess aid’s role in supporting the diffusion and spread of the adoption and institutionalization of specific rule of law institutions.

In Stage 2 we use an instrumental variable two‐way fixed‐effects panel regression approach to assess the effects of aid on several measures of the degree and quality of the rule of law within countries. This approach allows us to examine whether changes in aid levels to a country over time are associated with concomitant changes in the rule of law across society. Our panels are unbalanced and for the purposes of our analysis we lag the independent variable 2 years behind the outcome variable to allow time for aid to take effect. Thus, we predict rule of law measures 2 years following the investment of aid. Following Dreher and Langlotz (2017), we address the endogeneity of our aid measures using an instrument based on an interaction between the government fractionalization of donor countries and the probability of each recipient country to receive data from a given donor over the period of study. Applying Dreher and Langlotz’s approach, we first conduct a zero‐order OLS regression of each aid measure on the fractionalization‐probability interaction and then predict a fitted aid measure. This fitted aid measure is used as the excluded instrument to predict the first‐stage results in our instrumental variable regression models, and in the second‐stage two‐way fixed effects models we use the aid measures predicted in the first stage regression to model our rule of law measures. The instrument is outlined in the data section below.

### 3.2 Data

#### 3.2.1 Sample

Our population consists of the 161 countries listed in the AidData dataset (Research Release version 3.1) as having received aid at some point during the period of our study between 1995 and 2013. Owing to missing data on some covariates, our largest sample of countries in the event history analysis includes 154 of these countries, very closely approximating the population. In our panel regression analysis, the 154‐country sample is reduced in several models owing to limited data availability on the dependent variable. Still, with nearly all aid receiving countries in this period represented, we contend that our parameter estimates can—at least in the samples of 154 countries—be held to be close to a population estimate, and therefore we can focus less on the tests of statistical significance and more on the magnitude of the estimates.

#### 3.2.2 Outcome variables

To analyze the first dimension of the rule of law, our event history models estimate the risk of three separate legal institutional reform events. The first is the adoption of a new constitution or reinstatement of an existing constitution. Beyond constitutional reform, the first dimension of the rule of law also entails the creation or modification of laws. While there is a paucity of available cross‐national data on the passing of specific types of laws, we are nonetheless able to collect data pertaining to two significant categories of legislation: the occurrence of a reform to rape legislation (Frank et al., 2009, 2010); and the creation or amendment of counter‐terror legislation (Shor, 2011, 2017). We consider an evaluation of both rape and counter‐terror legislation as particularly instructive in assessing the influence of aid on World Society diffusion processes with respect to the rule of law, as they are proxies of three major donor priorities: gender equality, legal and judicial reform, and the securitization of aid (Brown & Grävingholt, 2016; Pickbourn & Ndikumana, 2016; Swiss, 2011, 2012).

To measure the second dimension of the rule of law, we use four different indicators. Among the two more general measures is the International Country Risk Guide’s (ICRG) “Law and Order” indicator, which measures the popular observance of the law and the strength and impartiality of legal institutions based on expert assessments (PRS Group, 2014). It ranges from 0 (a weak rule of law) to 6 (a strong rule of law). Second, we use the Worldwide Governance Indicators’ (WGI) aggregated “Rule of Law” index, which ranges from −2.5 (a weak rule of law) to 2.5 (a strong rule of law) (World Bank, 2018). The index is drawn from a variety of sources, including survey institutes, think tanks, non‐governmental organizations, international organizations, and private sector firms, and “captures perceptions of the extent to which agents have confidence in and abide by the rules of society, and in particular the quality of contract enforcement, property rights, the police, and the courts, as well as the likelihood of crime and violence” (World Bank, 2018).

The other two indicators are complementary in that they measure specific, but different, aspects of the second dimension of the rule of law—one is a proxy for security of private property, while the other is a proxy for security of the person. The first, Contract‐Intensive Money (CIM), measures the general confidence in the legal system’s capacity to enforce contracts and protect private property*.* CIM is a measure of how people choose to hold their financial assets—either as currency (i.e., cash) or in financial institutions as bank deposits or other financial instruments (Clague, Keefer, Knack, & Olson, 1999). In countries with a strong rule of law and trust in legal institutions, one would expect the vast majority of liquid assets to be held in financial institutions, where they are considered safe and also accumulate interest or other types of returns. Conversely, in countries with a weak rule of law, one would expect to find a stronger preference for currency even if it means forgoing interest income, as there is less confidence that money deposited in financial institutions is secure. The measure ranges from 0 to 1, reflecting the proportion of the total money supply held in financial institutions (International Monetary Fund, 2016).

Lastly, we use each country’s intentional homicide rate from the United Nations Office on Drug and Crime’s International Homicide Statistics database as a measure of both violence and legal compliance. The control of violence is arguably one of the most fundamental functions of a state (North, Wallis, & Weingast, 2009). As homicide is among the most severe manifestations of violence, the homicide rate can be considered an important indicator of the influence of law in society (i.e., the second dimension of the rule of law). Moreover, the homicide rate is considered the most reliable cross‐national measure of violence, as it is defined similarly across countries and it is more difficult to conceal relative to other types of violent crimes (Pinker, 2011).

Notably, the indicators of the first and second dimensions of the rule of law are not directly linked. That is, measures of constitutional or legislative change are independent from measures of the diffusion of the rule of law across society. As such, each of the two methodological stages result in separate, stand‐alone findings. The objective is not to directly test whether specific legal reforms result in specific society‐wide behavioral changes (which would be difficult due to current limitations of data availability). Rather, our interest lies in whether foreign aid has an influence on both the adoption of constitutional and legal reforms and on the overall strengthening of the rule of law. This strategy has the advantage of allowing a general assessment of the state of the rule of law in recipient countries.

#### 3.2.3 Explanatory variables: Foreign aid

Our focus in this study is to better understand the effects of foreign aid on the diffusion of rule of law institutions and norms. To assess these, we use three categories of aid flows in our analysis which measure aid at the aggregate level, narrow to a sectoral focus on conflict and security, and then to a specific focus on security sector reform and management. Our aid measures are derived from the AidData dataset Research Release version 3.1 (AidData, 2017; Tierney et al., 2011).

*Total ODA* measures official development assistance (ODA) received by a country from all sources in each year in millions of US dollars (USD). Median total aid in our largest sub‐sample of observations is $448 million.

The AidData dataset uses modified coding of the OECD Development Assistance Committee’s sectoral codes to classify aid spending. We use three of these sector codes to identify aid spending sub‐categories of interest:

1. *Total ODA to Conflict and Security* is all official development assistance received by a country coded in the AidData dataset as falling under the sector code for conflict, peace and security (code 15200 and its sub‐groups). Aid to conflict and security aid in our sample ranges from countries which receive no aid in a given year to a maximum of US$1.14 billion.
2. *Total ODA to Security Sector Reform (SSR) and Management* is a sub‐category of (1) above (code 15210). As mentioned, much of the aid in this category specifically targets rule of law institutions and their governance. The Development Assistance Committee describes this category as follows:
  Technical co‐operation provided to parliament, government ministries, law enforcement agencies and the judiciary to assist review and reform of the security system to improve democratic governance and civilian control; technical co‐operation provided to government to improve civilian oversight and democratic control of budgeting, management, accountability and auditing of security expenditure, including military budgets, as part of a public expenditure management programme; assistance to civil society to enhance its competence and capacity to scrutinise the security system so that it is managed in accordance with democratic norms and principles of accountability, transparency and good governance. (OECD, 2016)
  With this narrower focus, we expect aid in this category to have a stronger association with the measures of rule of law we examine in this study than aid generally. The median amount of aid to security sector reform in our largest sample is zero, reflecting that more than 50% of our sample countries receive no aid in support of security sector reform.
3. *Total Aid to Legal and Judicial Reform* (code 15130): Like (2) above, this category has a narrower focus on legal and judicial reform programs. The Development Assistance Committee describes this grouping as:
  Support to institutions, systems and procedures of the justice sector, both formal and informal; support to ministries of justice, the interior and home affairs; judges and courts; legal drafting services; bar and lawyers associations; professional legal education; maintenance of law and order and public safety; border management; law enforcement agencies, police, prisons and their supervision; ombudsmen; alternative dispute resolution, arbitration and mediation; legal aid and counsel; traditional, indigenous and paralegal practices that fall outside the formal legal system. Measures that support the improvement of legal frameworks, constitutions, laws and regulations; legislative and constitutional drafting and review; legal reform; integration of formal and informal systems of law. Public legal education; dissemination of information on entitlements and remedies for injustice; awareness campaigns. (OECD, 2016)
  Like aid to security sector reform above, we expect this category of aid to have a larger effect on both the adoption of legal reforms and the subsequent measures of rule of law. In our sample, the median level of annual aid to legal and judicial reform received by a country is approximately $570,000. To account for skewness in our aid measures, we take the log (base 2) of each measure. This means that in our regression analyses we can interpret the estimates as equivalent to a doubling of the aid measure, allowing for a simple conceptualization of the relative marginal effects of our logged measures. The untransformed summary statistics for our aid variables are shown in Table 1.^1^

**Table 1:** Summary statistics

	Mean	Median	Min	Max	*SD*
Aid measures (millions USD)					
Total ODA	1,483.4	447.9	0	63,232.6	3,910.3
ODA to conflict, peace, and security	13.4	0.25	0	1,135.6	66.5
ODA to SSR and management	2.72	0	−5.06	624.6	20.3
ODA to legal and judicial reform	10.1	0.57	−12.1	1,036.3	51.7
Controls					
GDP per capita (USD)	4,220.1	2089.4	50.0	60,290.2	6,823.2
Population (millions)	35.7	6.26	0.0092	1,357.4	140.9
Electoral democracy index	0.32	0.23	0.012	0.92	0.28
Gini coefficient	0.41	0.41	0.22	0.62	0.073
Government effectiveness	−0.35	−0.44	−2.27	1.57	0.69
Excluded population	0.15	0.090	0	0.87	0.20
INGO memberships	860.9	634	35	3,967	711.8
Human rights treaty ratifications	7.33	7	0	18	3.45
Dependent variables					
Intentional homicides	11.7	7.20	0.40	139.1	14.7
WGI rule of law index	−0.37	−0.46	−2.23	1.45	0.75
Contract‐intensive money	0.81	0.85	0.12	1.04	0.14
ICRG law and order index	3.42	3.50	0.50	6	1.13

#### 3.2.4 Controls

As both methodological stages examine separate types of outcomes, each has a unique suite of control variables. Descriptive statistics for these measures are shown in Table 1.

For the event history models in Stage 1, we control for factors that may influence the adoption of legal or constitutional reforms. This includes *national income* in the form of gross domestic product (GDP) per capita (logged for skewness), with a sample median of approximately $2,089 in constant 2005 USD (World Bank, 2018). To account for the effects of *democracy*, we use the Varieties of Democracy polyarchy index to measure changing levels of democracy in our sample countries over time (Coppedge et al., 2016). The polyarchy index ranges from 0 to 1, and our sample has a median of 0.23. We control for *state capacity* using the WGI Government Effectiveness measure (World Bank, 2018). Following the World Society literature (Tsutsui & Wotipka, 2004; Wotipka & Ramirez, 2008; Wotipka & Tsutsui, 2008), we also control for the influence of international non‐governmental organizations (INGOs) and human rights treaties. Our *INGO* measure counts the number of organizations in which a country’s citizens hold membership in a given year, compiled from the Yearbook of International Organizations (Union of International Associations, 2015). The median INGO memberships in our sample is 634. The *human rights treaty* measure counts the number of treaties a country has currently ratified in a given year, ranging from 0 to 18 in our sample (Office of the United Nations High Commissioner for Human Rights, 2018). Both the *INGO* and *human rights treaty* measure are logged to account for skewness.^2^ To account for country size, we include the log of the total *population* (World Bank, 2018). Countries in our largest sample range from a minimum population of 9,227 (Tuvalu in 1995) to nearly 1.36 billion (China in 2013). Finally, to ensure we are not simply picking up trends in rule of law institutions and measures over time, we include a *time trend* in our event history models.

In Stage 2, we include several control variables in our two‐way fixed‐effects analyses that have previously been shown to influence rule of law outcomes. Given that we examine several different measures of the dependent variable, we control for the primary factors identified in the literature as influencing the rule of law. However, we do not include all possible measure‐specific controls. While this strategy may increase the risk of omitted variable bias, we mitigate this risk by running two‐way fixed‐effects models, which control for all country‐specific time‐invariant (or slow changing) factors, and the effects of trends over time.

*Income inequality* is included as a control, as measured by the Gini coefficient (Solt, 2019). Following the logic of relative deprivation theory, higher levels of inequality are predicted to result in lower levels of the rule of law (Merton, 1938). Given the close ties between economic development and rule of law demonstrated in prior research (Dam, 2006; Haggard, MacIntyre, & Tiede, 2008; North, 1990), we include a *GDP per capita* (log) control as wealthier countries are expected to demonstrate higher levels of rule of law. As *democracy* is not a requisite of the rule of law as conceptualized here, we include it as a control. Past research suggests that democracy and the rule of law are mutually reinforcing (Rigobon & Rodrik, 2005); therefore, more democratic countries are expected to have a more effective rule of law.

As promoting the rule of law and maintaining the social order are core functions of the state (North et al., 2009), we control for *state capacity* using the *government effectiveness* measure cited above. State bureaucracies that are deemed low quality—that is, bureaucracies that lack institutional strength and political autonomy—are expected to be associated with comparatively lower levels of the rule of law.

Wimmer (2013) contends that *ethnic exclusion* from state power decreases the legitimacy of the state and the legal order and increases the propensity of ethnic conflict. In this vein, ethno‐political exclusion may lead to decreased levels of the rule of law. We therefore include it as a control, drawing upon an indicator that measures the proportion of the population belonging to politically relevant ethnic groups excluded from state power relative to the total population (Ethnic Power Relations Dataset, 2018; Vogt et al., 2015). Lastly, as with the Stage 1 models, we include a *population* (log) variable to account for country size.

#### 3.2.5 Instrument

To account for the endogeneity of our aid measures and for the potential of reverse causality, we create an excluded instrument using an interaction of government fractionalization in donor countries (the chance that a random draw of two members of the donor government represent different political parties) (World Bank, 2018) and the probability of each recipient country to receive aid from each donor in the AidData dataset in a given year (Dreher & Langlotz, 2017). Dreher and Langlotz (2017) demonstrate this instrument as a strong predictor of aid flows unrelated to recipient country characteristics because it reflects likely increased aid budgets in donor countries with fractionalized governments as a means of appeasing coalition members. We calculate this interaction for each donor–recipient dyad pair in the AidData dataset, and then include the mean fractionalization‐probability value for each recipient country as the predictor of our fitted aid measure in our instrumental variable regression models below.

## 4 RESULTS

### 4.1 Event history results

The results of our first stage of analysis (event history models of rule of law institutional adoption) are shown in Table 2. Each model shows the hazard ratio estimates for the adoption of our three legal institutional reforms predicted by each of the categories of aid (Stare & Maucort‐Boulch, 2016).^3^

**Table 2:** Event history regression of rule of law institutions on foreign aid

	Constitutional reforms				Rape law reforms				Terror law reforms			
	(1)	(2)	(3)	(4)	(5)	(6)	(7)	(8)	(9)	(10)	(11)	(12)
Aid measures												
Total aid	1.062				1.112				1.061*			
Conflict aid		1.062				1.210*				1.004		
SSR aid			1.142				1.421**				1.021	
Legal/judicial aid				1.053				1.178				1.079*
Controls												
Logged population	1.049	1.077	1.087	1.081	0.661*	0.692*	0.683*	0.706*	0.942	0.980	0.960	0.968
Logged GDP per capita	0.949	0.942	0.934	0.941	0.895	0.879	0.854	0.892	1.009	0.983	0.985	0.996
Electoral democracy index	2.614*	2.559*	2.747**	2.545*	2.207	2.145	2.235	2.041	1.319	1.256	1.298	1.271
Government effectiveness	0.555*	0.588*	0.589*	0.571*	1.287	1.378	1.370	1.358	1.339*	1.325*	1.365*	1.356*
Logged INGO memberships	0.864	0.882	0.853	0.871	1.858	2.006*	2.066*	1.829	1.272*	1.285*	1.347**	1.279*
Logged human rights treaties	1.187	1.194	1.200	1.210	1.268	1.237	1.195	1.355	1.122	1.184	1.142	1.151
Year	0.948*	0.942*	0.937*	0.944*	0.832***	0.810***	0.816***	0.817***	0.993	0.987	0.997	0.983
Country‐years	2,800	2,800	2,726	2,798	2,800	2,800	2,726	2,798	2,800	2,800	2,726	2,798
Countries	154	154	154	154	154	154	154	154	154	154	154	154
Failures	78	78	77	78	37	37	37	37	322	322	318	322
Log likelihood	−198	−197	−194	−198	−117	−116	−114	−117	−274	−277	−266	−274
AIC	413	413	406	413	253	251	246	252	566	572	550	566

Note

Exponentiated coefficients (hazard ratios).

* *p* < .05;

** *p* < .01;

*** *p* < .001.

Models 1–4 in Table 2 show the predicted effect of our aid measures on the adoption of constitutional reforms. While none of the four aid measures attains a *p* value below .05, each estimate shows that a doubling of that aid type predicts between a 5.3% (legal/judicial aid) and 14.2% (security sector reform aid) increase in the risk of constitutional reforms. The controls affect the adoption of constitutional reforms differently: government effectiveness predicts a lower chance of constitutional reforms, while higher levels of democracy predict an increased chance of adoption. The chance of constitutional reforms declines with time, between 5.2% and 6.3% a year.

Like the constitutional reforms, the adoption of rape law reforms modeled in Models 5–8 in Table 2 show that all aid categories predict an increased risk of rape law reforms, and the estimates for both conflict aid and security sector reform aid attain *p* values below the typical .05 threshold. The magnitude of these significant effects varies from a 21% increase (Conflict aid) to a 42% increase (security sector reform aid) in the chance of rape law reform. The control variables reveal that more populous countries have a lower likelihood of reforming their rape laws, while countries with more INGO memberships appear to have in some cases more than double the chance of these reforms than countries less plugged into the global INGO network.

Finally, in Models 9–12 of Table 2, we report the results of the event history models of the effect of all four aid measures on terror law adoption. Here, total aid and legal/judicial aid is associated with the adoption of terror laws. In Model 9, a doubling of total aid predicts a more than 6% increase (*p* < .05) in the risk of terror law adoption, while Model 12 shows that a doubling of legal/judicial aid predicts an almost 8% increase (*p* < .05). The direction of the hazard ratios for conflict aid and security sector reform aid also suggest increased risk of reforms but to a lesser degree; we fail to reject the null hypothesis in both cases. When it comes to terror law adoption, our control variables show that states with more effective governments and a greater number of INGO membership ties are more likely to adopt terror law reforms.

**Figure 2:**
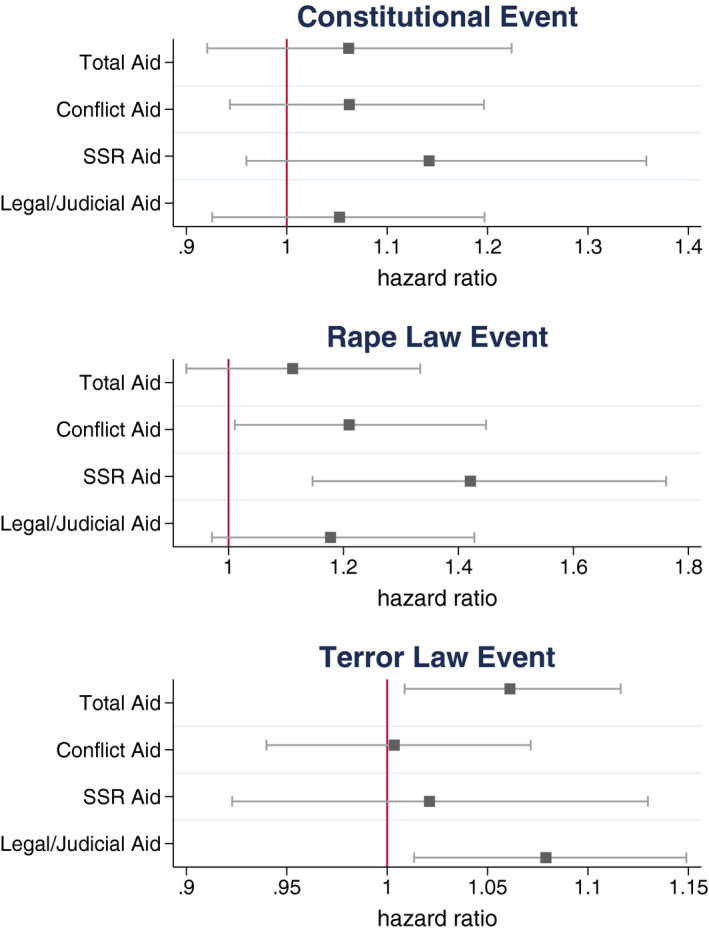
Predicted effect of doubling of aid amounts on risk of rule of law institution event [Colour figure can be viewed at wileyonlinelibrary.com]

Figure 2 compares hazard ratio estimates for each category of aid across each institutional reform event. In both the constitutional and rape law reform cases, security sector reform aid has a larger effect on the risk of an event than aid to conflict and security generally, and in two of the three cases is a stronger predictor of rule of law reforms than either total aid or legal and judicial aid. Yet, in the case of terror law reforms our results show that this pattern is reversed and both total aid and legal/judicial aid are much stronger predictors of adoption. These results show support for our first hypothesis and partial support for our third hypothesis on the stronger effects of security sector reform and legal/judicial aid.

### 4.2 Instrumental variable two‐way fixed effects results

Stage 2 of our analysis estimates the effects of our four categories of foreign aid on each of our four measures of rule of law (homicide rate, contract‐intensive money, and the two rule of law indices). In Table 3, we show the results of our zero‐order OLS regression of our aid measures on an interaction of donor government fractionalization and the joint probability of a recipient country to receive aid from all donors in a given year. The fitted aid measure predicted from these models for each country‐year observation is used as the excluded instrument in the first stage of our instrumental variable models reported in Tables 4, 5, 6, 7. The final instrumental variable results are reported in the second‐stage portion of each table, and include test results for identification of weak instruments following Stock and Yogo (2005).

**Table 3:** Zero‐order regression results (OLS) for instrumental variable regressions

	(1)	(2)	(3)	(4)
	Total aid	Conflict aid	SSR aid	Legal/judicial aid
Lagged aid measures	0.760***	0.806***	0.611***	0.606***
Lagged donor fractionalization–recipient probability interaction	4.383***	1.523***	0.875***	2.562***
Constant	0.353***	−0.206**	−0.078	−0.318***
Observations	2,864	2,864	2,768	2,860
*R* ^2^	0.78	0.68	0.36	0.47
Country fixed effects	No	No	No	No
Year fixed effects	No	No	No	No

* *p* < .05;

** *p* < .01;

*** *p* < .001.

**Table 4:** Fixed‐effects instrumental variable regression of homicide rate on foreign aid

Second‐stage regression results (two‐way FE)				
	(1)	(2)	(3)	(4)
	Total aid	Conflict aid	SSR aid	Legal/judicial aid
Aid measures				
Total aid	−0.406			
Conflict aid		−1.423**		
SSR aid			−2.799	
Legal/judicial aid				−1.152
Controls				
Logged population	6.916*	7.812*	9.885*	9.089*
Logged GDP per capita	−1.137	−1.179	−0.564	−0.307
Electoral democracy index	−0.689	−0.526	−0.731	−0.237
Gini coefficient	−3.731	2.467	−4.116	−6.464
Government effectiveness: Estimate	−5.319***	−4.929***	−4.065**	−4.844***
Excluded population	3.559	3.329	2.439	1.922
Observations	925	925	910	923
Countries	88	88	88	88
*R* ^2^	0.06	−0.00	−0.23	−0.05
Country fixed effects	Yes	Yes	Yes	Yes
Year fixed effects	Yes	Yes	Yes	Yes
Cragg–Donald weak identification	113.87	101.13	11.01	17.68
Anderson underidentification test	102.50	92.29	11.14	17.75
First‐stage regression results (two‐way FE)				
Fitted aid measures	0.498***	0.402***	0.192***	0.240***
Logged population	3.003***	1.260	1.581*	3.124***
Logged GDP per capita	0.436	0.038	0.242	0.846*
Electoral democracy index	−0.979^*^	−0.178	−0.170	−0.046
Gini coefficient	−2.099	3.018	0.534	−2.296
Government effectiveness: Estimate	−0.097	0.102	0.341	0.285
Excluded population	−0.511	−0.216	−0.557	−1.481
Observations	925	925	910	923
Countries	88	88	88	88
*R* ^2^	0.18	0.25	0.13	0.11
Country fixed effects	Yes	Yes	Yes	Yes
Year fixed effects	Yes	Yes	Yes	Yes

* *p* < .05;

** *p* < .01;

*** *p* < .001.

**Table 5:** Fixed‐effects instrumental variable regression of rule of law index (WGI) on foreign aid

Second‐stage regression results (two‐way FE)				
	(1)	(2)	(3)	(4)
	Total aid	Conflict aid	SSR aid	Legal/judicial aid
Aid measures				
Total aid	0.008			
Conflict aid		0.012		
SSR aid			0.083**	
Legal/judicial aid				0.026
Controls				
Logged population	0.042	0.059	−0.112	0.007
Logged GDP per capita	0.150***	0.157***	0.153***	0.140***
Electoral democracy index	−0.075	−0.081*	−0.121**	−0.079*
Gini coefficient	0.848*	0.806*	0.555	0.973**
Government effectiveness	0.406***	0.403***	0.383***	0.392***
Excluded population	−0.150**	−0.146**	−0.131*	−0.140**
Observations	1688	1688	1643	1686
Countries	135	135	133	135
*R* ^2^	0.23	0.22	0.04	0.21
Country fixed effects	Yes	Yes	Yes	Yes
Year fixed effects	Yes	Yes	Yes	Yes
Cragg–Donald weak identification	306.73	331.63	50.93	41.87
Anderson underidentification test	258.92	276.20	49.91	41.29
First‐stage regression results (two‐way FE)				
Fitted aid measures	0.540***	0.539***	0.299***	0.273***
Logged population	2.789***	0.793*	1.370***	2.277***
Logged GDP per capita	0.050	−0.320	−0.089	0.360
Electoral democracy index	−0.074	0.127	0.259	0.004
Gini coefficient	0.263	0.425	3.292	−4.852
Government effectiveness	−0.264	−0.061	0.117	0.376
Excluded population	−0.083	−0.479	−0.297	−0.428
Observations	1688	1688	1643	1686
Countries	135	135	133	135
*R* ^2^	0.24	0.33	0.14	0.14
Country fixed effects	Yes	Yes	Yes	Yes
Year fixed effects	Yes	Yes	Yes	Yes

* *p* < .05;

** *p* < .01;

*** *p* < .001.

**Table 6:** Fixed‐effects instrumental variable regression of contract‐intensive money on foreign aid

Second‐stage regression results (two‐way FE)				
	(1)	(2)	(3)	(4)
	Total aid	Conflict aid	SSR aid	Legal/judicial aid
Aid measures				
Total aid	0.006*			
Conflict aid		−0.001		
SSR aid			0.011	
Legal/judicial aid				0.017
Controls				
Logged population	−0.025	0.001	0.012	−0.014
Logged GDP per capita	0.039***	0.039***	0.036***	0.038***
Electoral democracy index	0.010	0.009	0.005	0.015
Gini coefficient	0.041	0.045	0.157	0.066
Government effectiveness	0.046***	0.045***	0.045***	0.032*
Excluded population	−0.035*	−0.041**	−0.036*	−0.037*
Observations	1,354	1,354	1,335	1,354
Countries	124	124	123	124
*R* ^2^	0.19	0.20	0.16	0.03
Country fixed effects	Yes	Yes	Yes	Yes
Year fixed effects	Yes	Yes	Yes	Yes
Cragg–Donald weak identification	193.29	206.52	23.19	8.40
Anderson underidentification test	169.67	179.58	23.17	8.49
First‐stage regression results (two‐way FE)				
Fitted aid measures	0.505***	0.475***	0.238***	0.140**
Logged population	3.280***	−0.371	−0.289	0.889
Logged GDP per capita	−0.081	−0.415	0.016	0.066
Electoral democracy index	−0.052	0.397	0.324	−0.367
Gini coefficient	0.276	1.024	−0.604	−1.297
Government effectiveness	−0.088	−0.079	0.146	0.725**
Excluded population	−0.510	−0.207	−0.314	−0.179
Observations	1,354	1,354	1,335	1,354
Countries	124	124	123	124
*R* ^2^	0.21	0.32	0.13	0.11
Country fixed effects	Yes	Yes	Yes	Yes
Year fixed effects	Yes	Yes	Yes	Yes

* *p* < .05;

** *p* < .01;

*** *p* < .001.

**Table 7:** Fixed‐effects instrumental variable regression of law and order index (ICRG) on foreign aid

Second stage regression results (two‐way FE)				
	(1)	(2)	(3)	(4)
	Total aid	Conflict aid	SSR aid	legal/judicial aid
Aid measures				
Total aid	−0.000			
Conflict aid		0.076***		
SSR aid			0.106*	
Legal/judicial aid				0.165**
Controls				
Logged population	0.285	0.180	0.363*	−0.190
Logged GDP per capita	−0.140*	−0.099	−0.105	−0.208*
Electoral democracy index	−0.117	−0.140	−0.113	−0.079
Gini coefficient	3.056***	2.759**	2.460*	3.564***
Government effectiveness	0.256***	0.266***	0.203**	0.163
Excluded population	−0.182	−0.129	−0.152	−0.066
Observations	1,485	1,485	1,443	1,483
Countries	103	103	101	103
*R* ^2^	0.17	0.16	0.15	−0.03
Country fixed effects	Yes	Yes	Yes	Yes
Year fixed effects	Yes	Yes	Yes	Yes
Cragg–Donald weak identification	317.62	311.51	80.42	36.64
Anderson underidentification test	261.65	257.56	77.07	36.26
First‐stage regression results (two‐way FE)				
Fitted aid measures	0.591***	0.539***	0.397***	0.271***
Logged population	2.711***	0.872*	1.066**	2.459***
Logged GDP per capita	0.259	−0.375	−0.137	0.306
Electoral democracy index	−0.272	0.202	0.203	−0.203
Gini coefficient	−2.213	2.272	2.731	−3.535
Government effectiveness	−0.720**	−0.167	0.169	0.484
Excluded population	−0.263	−0.499	0.017	−0.568
Observations	1,485	1,485	1,443	1,483
Countries	103	103	101	103
R^2^	0.28	0.35	0.17	0.14
Country fixed effects	Yes	Yes	Yes	Yes
Year fixed effects	Yes	Yes	Yes	Yes

* *p* < .05;

** *p* < .01;

*** *p* < .001.

**Figure 3:**
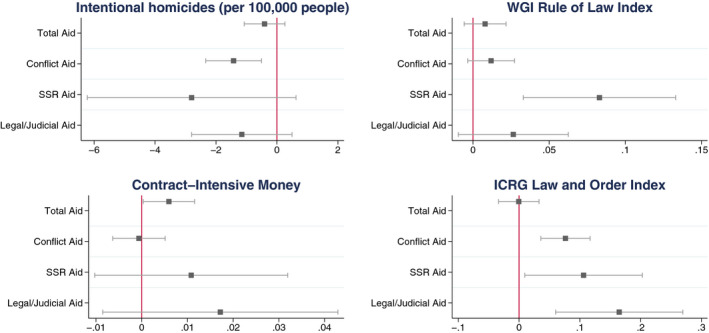
Predicted effect of doubling of aid amounts on change in rule of law measures [Colour figure can be viewed at wileyonlinelibrary.com]

Predicted marginal effects of our aid measures in the analyses from Tables 4, 5, 6, 7, are also shown in Figure 3. Because our aid measures are logged using Base 2, we can interpret the coefficients for each aid measure in these models as the marginal effect of a doubling of aid on the dependent variable.

Although aid generally shows a beneficial relationship to the rule of law in terms of direction of relationship with each of the aid measures, the magnitude and tests of statistical significance for many of these estimates suggests that this relationship is tenuous at best and modest in size. The association of aid to improved rule of law is clearest in the ICRG Law and Order Index. Still, even the 0.165 point increase on this index predicted by a doubling of legal/judicial aid is meager compared to a standard deviation for the ICRG Law and Order index of 1.13 in our sample. Thus, although our results indicate aid is in some cases associated at statistically significant levels with improved rule of law in terms of our various metrics, there is not robust and convincing support for our Hypothesis 2 in this respect, and security sector reform‐related and legal/judicial‐related aid do not appear to be consistently more beneficial to rule of law than either of the other categories of aid, except in the case of the ICRG Law and Order measure. Indeed, for the homicide measure, only total conflict aid is significantly associated with decreasing homicide rates, while for Contract‐Intensive Money, only total aid predicts a significantly significant increase.

The instrumental variable regression results from Stage 2 of our analysis suggest that the ability of aid to promote the rule of law, beyond the diffusion of certain institutional forms or reforms, may be limited and points to potential decoupling of rule of law institutional reforms and practices that aid cannot surmount and to which it may, in fact, contribute. We examine this decoupling in the next section of the paper.

## 5 DISCUSSION

In this paper we make two primary contributions. First, we provide evidence of the relationship of aid to the diffusion of some common rule of law institutions. Second, we demonstrate that aid appears to play a larger role in the isomorphism of state institutions than it does in strengthening states’ performance of them (i.e., states’ legal reach). Moreover, in analyzing both dimensions of the rule of law, our study addresses a gap in the World Society and law literature, which “mostly overlooks implementation … [and] attends almost entirely to law on the books” (Halliday & Carruthers, 2007, p. 1139).

The results suggest that aid is more strongly associated with the first dimension of the rule of law (i.e., legal institutional reforms) than with the second dimension (i.e., rule of law outcomes). These findings are both consistent with, and contribute to, the rule of law literature. The results suggest that Cravo’s (2016) case study findings of the link between aid and legislative reforms is generalizable across a large number of countries. However, the findings differ from the extant literature in suggesting that targeted aid—specifically security sector reform aid—does have a positive (even if small) impact on certain aspects of the rule of law. Moreover, total official development assistance is found to have virtually no effect on the rule of law, unlike previous studies that found a significant negative effect (Knack, 2001; Rajan & Subramanian, 2007; Young & Sheehan, 2014). Taken together, the analyses of various categories of aid directly targeting the rule of law reveal that aid may be somewhat more effective—or less deleterious—in strengthening the rule of law than found in prior studies. As such, the results also reinforce the importance of disaggregating total official development assistance for a sectoral analysis of the effectiveness of rule of law aid.

As aid appears less harmful and more helpful to rule of law outcomes than originally thought, these results could be interpreted as supportive of the current approach to rule of law reforms. However, given aid’s limited impact on the second dimension of the rule of law, in our view the results tend to lend support to critics of the current approach. These critics contend that a legal system cannot simply be transferred from one country to another, as the rule of law involves far more than getting a particular legal framework in place (Carothers, 2009; Erbeznik, 2018; Haggard et al., 2008). Rather, “[i]t is a transformative process that changes how power is both exercised and distributed in a society … [and] also involves basic changes in how citizens relate to state authority and also to one another” (Carothers, 2009, pp. 59, 60). In corroborating this perspective, our results imply that state‐centric legal reforms are likely to have limited success, as they are insufficient in significantly influencing the second dimension of the rule of law. This premise has led to the proposal of “second generation” rule of law assistance to overcome these limitations (Kleinfeld, 2012). These rule of law reforms are meant to go beyond justice institutions and have a broader focus on state‐society relations, local norms, and political culture, potentially increasing the effectiveness of aid on rule of law outcomes.

When considering aid’s role in World Society more generally, our results support the idea that sector‐focused foreign aid can influence the diffusion/adoption of common models by states. This is in keeping with our hypotheses, and with earlier research on aid from a World Society perspective which theorizes a diffusion role for aid (Swiss, 2016b, 2016c). Still, our evidence suggests that aid’s influence on isomorphism may have its limits when it comes to supporting not only the spread of models, but their institutionalization and efficacy. Consequently, foreign aid may augment the discrepancy between the law on the books and the law in practice—a finding that corroborates Halliday and Carruthers’ (2007) study of corporate bankruptcy laws. Though this may be a finding specific to rule of law, if replicated in other studies of sector‐focused aid, it would point to a potential limitation of World Society processes in promoting development. Indeed, we may find evidence to suggest that aid’s role in supporting diffusion far exceeds that of supporting implementation of common models and practices among states, with aid potentially contributing to—rather than lessening—the decoupling between state institutional structure and practice.

Future research should explore the aid–rule of law relationship more closely to understand the micro‐level dynamics of how aid initiatives support the diffusion of these approaches, as well as to better understand the reasons why aid’s influence appears to diminish when it comes to the promotion of the rule of law. Additional case studies of specific security sector reform and rule of law initiatives would be useful future research in this regard. Likewise, as only a single sector‐focused study of the role aid plays in diffusing World Society models, researchers should look to investigate the role of aid in diffusion of other world cultural norms around environment, human rights, education, and more. Only by intensifying the examination of foreign aid’s role in the diffusion and enactment processes described by World Society theory will we get a fuller sense of how aid shapes isomorphism among aid recipient states.

## Data Availability

The data that support the findings of this study are available from the corresponding author upon reasonable request.

## NOTES

^1^ While many studies of the effects of foreign aid consider aid flows in terms of per capita aid received, or as a percentage of a country’s annual GDP, we specify aid as the aggregate annual flow because there is no theoretical justification to do otherwise given the nature of the institutional adoption and institutionalization process we are modeling (see Uslaner, 1976). Aid directed at such reforms would neither be proportional to the size of a country’s population nor its economy, and arguably could be relatively consistent in cost across different country contexts. Furthermore, bilateral aid donors seldom make aid allocations based on a per capita or percentage of GDP basis, focusing instead on funding specific projects which target specific recipient country or donor priorities.

^2^ As with our aid measures, we add one to account for zeros and then take the log (base 2) of each logged control variable.

^3^ Hazard ratios (HR) represent the risk of an event occurring at a given point in time given a one unit change in the variable. An HR above one represents an increased risk, while an HR below one indicates reduced risk (Stare & Maucort‐Boulch, 2016).
